# Striatal morphology correlates with frontostriatal electrophysiological motor processing in Huntington's disease: an IMAGE‐HD study

**DOI:** 10.1002/brb3.511

**Published:** 2016-07-27

**Authors:** Lauren M. Turner, David Jakabek, Fiona A. Wilkes, Rodney J. Croft, Andrew Churchyard, Mark Walterfang, Dennis Velakoulis, Jeffrey C. L. Looi, Nellie Georgiou‐Karistianis, Deborah Apthorp

**Affiliations:** ^1^ Research School of PsychologyCollege of Medicine, Biology, & EnvironmentAustralian National UniversityCanberraAustralian Capital TerritoryAustralia; ^2^ Graduate School of MedicineUniversity of WollongongWollongongNew South WalesAustralia; ^3^ Academic Unit of Psychiatry and Addiction MedicineAustralian National University Medical SchoolCanberra HospitalCanberraAustralian Capital TerritoryAustralia; ^4^ School of Psychology & Illawarra Health & Medical Research InstituteUniversity of WollongongWollongongNew South WalesAustralia; ^5^ School of Psychological SciencesFaculty of Medicine, Nursing and Health SciencesMonash UniversityMonashVictoriaAustralia; ^6^ Calvary Health Care Bethlehem HospitalCaulfieldVictoriaAustralia; ^7^ Neuropsychiatry UnitRoyal Melbourne Hospital, and Melbourne Neuropsychiatry CentreUniversity of MelbourneMelbourneVictoriaAustralia

**Keywords:** Compensation, Huntington's disease, morphology, motor, motor response potentials, striatum, structural

## Abstract

**Background:**

Huntington's disease (HD) causes progressive atrophy to the striatum, a critical node in frontostriatal circuitry. Maintenance of motor function is dependent on functional connectivity of these premotor, motor, and dorsolateral frontostriatal circuits, and structural integrity of the striatum itself. We aimed to investigate whether size and shape of the striatum as a measure of frontostriatal circuit structural integrity was correlated with functional frontostriatal electrophysiological neural premotor processing (contingent negative variation, CNV), to better understand motoric structure–function relationships in early HD.

**Methods:**

Magnetic resonance imaging (MRI) scans and electrophysiological (EEG) measures of premotor processing were obtained from a combined HD group (12 presymptomatic, 7 symptomatic). Manual segmentation of caudate and putamen was conducted with subsequent shape analysis. Separate correlational analyses (volume and shape) included covariates of age, gender, intracranial volume, and time between EEG and MRI.

**Results:**

Right caudate volume correlated with early CNV latency over frontocentral regions and late CNV frontally, whereas right caudate shape correlated with early CNV latency centrally. Left caudate volume correlated with early CNV latency over centroparietal regions and late CNV frontally. Right and left putamen volumes correlated with early CNV latency frontally, and right and left putamen shape/volume correlated with parietal CNV slope.

**Conclusions:**

Timing (latency) and pattern (slope) of frontostriatal circuit‐mediated premotor functional activation across scalp regions were correlated with abnormalities in structural integrity of the key frontostriatal circuit component, the striatum (size and shape). This was accompanied by normal reaction times, suggesting it may be undetected in regular tasks due to preserved motor “performance.” Such differences in functional activation may reflect atrophy‐based frontostriatal circuitry despecialization and/or compensatory recruitment of additional brain regions.

## Introduction

Huntington's disease (HD) is a progressive, autosomal dominant disease which results in widespread degeneration of cortical gray and white matter, as well as localized atrophy of the striatum (Vonsattel et al. 1985; Hobbs et al. 2010). Although symptom onset typically begins around age 40 (Walker 2007), neuroimaging demonstrates progressive atrophy up to 20 years prior (Tabrizi et al. 2009, 2011, 2013; van den Bogaard et al. 2011a,b; Aylward et al. 2012; Dominguez et al. 2013; Georgiou‐Karistianis et al. 2013). Involuntary motor dysfunction is a disease hallmark, reflecting damage to the basal ganglia. Symptomatic (symp‐HD) individuals present with motor symptoms such as chorea (Nance et al. 2011) as well as detriment to initiation, execution, and termination of voluntary movement (Smith et al. 2000; Quinn et al. 2001; Boulet et al. 2005; Lemay et al. 2008). Despite neuronal degeneration, individuals in early stages (pre‐HD) present with minimal functional differences compared with healthy persons (e.g., they react quickly, and can perform simple tasks at the same speed). However, impairments have been detected during more complex motor tasks (e.g., Farrow et al. 2006; Georgiou‐Karistianis et al. 2014b), or in subcomponents of performance efficiency (e.g., increased tapping variability) within larger studies (e.g., Bechtel et al. 2010). Retention of motor functionality in pre‐HD in the context of progressive atrophy is not well understood. One suggestion implicates functional connectivity: additional brain regions are recruited through reorganization of frontostriatal circuitry to counter early structural changes, compensatory processes which may fail with additional task demands and greater atrophy (Beste et al. 2007, 2009; Klöppel et al. 2009; Dominguez et al. 2013; Georgiou‐Karistianis et al. 2013, 2014a; Koenig et al. 2014). Electroencephalography (EEG) shows promise in identifying subtle changes in integrity of motor processing, and may offer new insights into the mismatch between neural atrophy and successful motor performance (Turner et al. 2015).

Electroencephalography has been suggested to be more sensitive to early disruption of cortical connectivity in HD than typical clinical measures (Lefaucheur et al. 2002), and shows promise in providing a measure of disease progression prior to observable motor deficits. EEG allows for the measurement of neural activity associated with sensorimotor integration and motor planning via the contingent negative variation (CNV; Ikeda et al. 1997). Such premotor function reflects cortical sources including the supplementary motor area (SMA; Macar et al. 2004), primary motor area (M1), and basal ganglia (Rektor et al. 2004). The early component of the CNV indexes prefrontal and supplementary sensorimotor areas (SSMA), the late component the prefrontal, M1, primary sensory, temporal, and occipital and SSMA areas (Hamano et al. 1997). As an index of complex premotor‐related activation, the CNV may be capable of detecting subtler changes in motor processing resulting from changing structural integrity. For example, we recently demonstrated abnormal CNV in pre‐HD (early, overengagement of premotor processes that were less focused than controls and occurred across electrode sites), despite intact motor processing (as measured by the readiness potential) and normal reaction times (Turner et al. 2015). This irregular CNV activation may reflect changes in functional connectivity.

**Figure 1:**
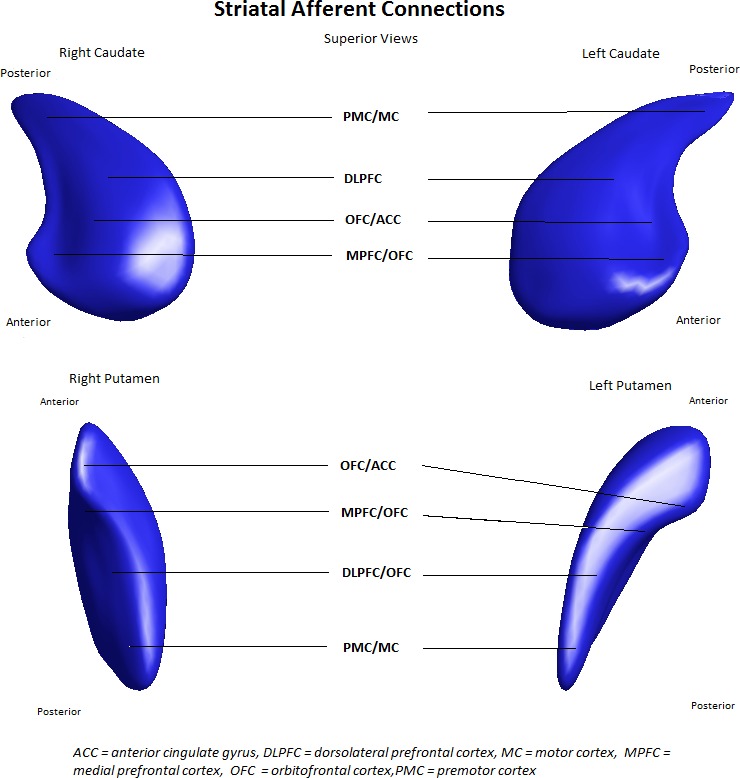
Striatal afferent connections indicate surface regions of the striatum which receive afferents from respective regions of cortex. Reproduced with permission from Looi et al. (2011).

Premotor and motor activation rely on parallel and converging circuitry traversing both brain hemispheres (Alexander et al. 1989). The striatum represents a critical node in a number of fronto‐striato‐pallido‐thalamo‐cortical re‐entrant circuits which regulate cognitive, emotional, and behavioral as well as motor functions (Draganski et al. 2008). Based on the frontostriatal circuit model (Alexander et al. 1986), a number of studies have shown preferential localization of motor control to the dorsolateral prefrontal cortex (DLPFC) and premotor and motor loop circuits (e.g., Taniwaki et al. 2003; Lehéricy et al. 2004; Utter and Basso 2008). The DLPFC can be thought of as a “cognitive loop,” which underpins executive functioning (Alexander et al. 1986; Cummings 1993). The striatum is highly topographically organized based on afferents from the cortex (Bohanna et al. 2011; see Fig. 1). Structural changes in the striatum are thus one possible mechanism by which the CNV may be affected in HD, in that loss of structural integrity may disrupt frontostriatal circuits involved in motor control, prior to the onset of clinical motor sequelae. One avenue of research that may clarify this issue is by linking electrophysiological measures of premotor activation (CNV) with structural MRI measures of striatal morphology.

Recently, functional magnetic resonance imaging (fMRI) studies have implicated functional connectivity changes in pre‐HD, including frontostriatal networks implicated in motor control (Unschuld et al., 2012; Georgiou‐Karistianis et al. 2013; Georgiou‐Karistianis et al. 2014b; Koenig et al. 2014; Poudel et al. 2014, 2015). Weakened M1 connectivity with SMA and DLPFC was found only in pre‐HD individuals temporally distant from onset of symptoms (Wolf et al. 2012), suggesting that reorganization may be a dynamic process depending on disease stage and the availability of neural resources. Klöppel et al. (2009) identified increased compensation in pre‐HD individuals, involving flexible recruitment of premotor and parietal areas, dependent on the pace and complexity of motor sequences. Reduced primary motor activation has been accompanied by parietal overactivation in symp‐HD (Bartenstein et al. 1997; Weeks et al. 1997; Gavazzi et al. 2007) and by excessive thalamo‐cortical activation during motor sequence processing in presymptomatic individuals (pre‐HD; Feigin et al. 2006). Furthermore, reduced sensorimotor network synchrony occurs prior to cognitive impairments in dorsal networks and has also been associated with performance deficits, such as imprecision during speeded self‐tapping (Poudel et al. 2014). Reduced connectivity of M1 both within and outside the motor network has been associated with reduced motor performance, greater motor symptoms, and neostriatal atrophy, and may reflect compensatory reorganization of function as well as decreased preferential connectivity (specialization) in pre‐HD (Koenig et al. 2014). Combining an electrophysiological measure of premotor activation (CNV) with structural MRI will enable investigation of subtle components of motor response, and their relationship with striatal morphology. A better understanding of the structural–functional integrity specific to the neural circuitry impaired during the disease will inform our understanding of the function and pathophysiology of HD, such as possible network despecialization/compensation through progressive atrophy.

In this study, we sought to explore neural substrates of abnormal premotor activation in HD (as measured by the CNV) identified in our previous paper (Turner et al. 2015). There are two key reasons to investigate the striatum, based on putative alterations in frontostriatal circuit structure and function, as reflected by CNV. First, there is a neuroanatomical rationale, based on striatal atrophy in HD and the localization of frontostriatal motor afferents to the caudate head and body, as well as the medial and lateral aspects of the putamen (Haber 2003; Draganski et al. 2008). This rationale predicts that disease progression will undermine frontostriatal motor connectivity. Second, there is preliminary evidence that changes in frontostriatal functional connectivity occur early in disease processes and may enable compensation. Hence, integrative examination of structure–function correlations may yield new insights into mechanisms of maintenance of function. Based on the preceding, we hypothesized: (1) that an abnormal CNV would be associated with reduced structural integrity of the key frontostriatal circuit components, caudate and putamen, as measured by morphology (Looi and Walterfang 2013); and (2) as the CNV requires premotor and motor activation, morphometry (quantification of the shape) of the striatum will likely demonstrate atrophy (shape deflation) in the lateral posterior caudate and putamen structures, reflecting reduced functional connectivity of the dorsolateral prefrontal, motor, and premotor frontostriatal circuits.

In order to investigate our hypotheses, both CNV and structural MRI data were obtained from a group of HD participants. To maximize statistical power and account for within‐group variability in disease burden score (DBS), cytosine–adenine–guanine (CAG) repeats, and disease onset, we included both presymptomatic (pre‐HD) and symptomatic individuals (symp‐HD) in our sample. We hypothesized that increased CNV amplitude, representing neural premotor processing during a sensorimotor integration task, would be associated with smaller volumes (indicating more atrophy) in the caudate and putamen.

## Methods

### Acquisition procedures

#### Participants

Participant data were obtained in 2008–2009 as part of the IMAGE‐HD project (Georgiou‐Karistianis et al. 2013), with EEG and MRI data collected on different days. Time periods between EEG testing and MRI were computed for each individual (“time to scan”), and used as a covariate in all analyses. Participants consisted of 7 genetically confirmed HD individuals (five males; symp‐HD) and 12 genetically confirmed prodromal individuals with no clinical manifestations (seven males; pre‐HD). This represents the entire HD sample reported in Turner et al. (2015) that also completed the imaging portion of the study (and that had usable data).^1^ All participants provided consent to participate in EEG and imaging studies and were assessed by a neurologist (A. C.). Ethics approval was obtained from the Melbourne Health Human Research Ethics Committee and for image analysis, the ANU Human Research Ethics Committee. Research procedures complied with the Code of Ethics of the World Medical Association (Declaration of Helsinki).

#### Clinical, neuropsychiatric, and cognitive measures

In addition to EEG and MRI data collection, a range of clinical, neuropsychiatric, and cognitive measures were collected. Participant demographics and clinical data are displayed in Table 1; only measures relevant to the present paper are reported below. We display between‐group data to show subgroup characteristics are similar to those reported in the literature, and discuss these briefly here. In order to characterize the groups, HD individuals with an UHDRS [Unified Huntington's Disease Rating Scale (1996)] total motor score of 5 or less were included in the pre‐HD group and those with scores greater than 5 in the symp‐HD group (Tabrizi et al. 2011). Nonparametric Mann–Whitney *U* tests indicate that as expected, symp‐HD had right and left caudate and putamen volumes that were significantly smaller than pre‐HD. Symp‐HD were also significantly older (*P *=* *0.045), and scored significantly more poorly on Trails B (*P *=* *0.005; 158.00 and 73.96 msec), speeded finger tapping (*P *<* *0.001; 335.53 and 215.86), reaction time during the CNV task (*P *<* *0.05; 1272.94 and 820.17), and the UHDRS total motor score (*P *<* *0.001). These results are as expected given the level of disease progression in these individuals. As our aim was to combine genetically confirmed individuals into one group for increased power, between‐group data will not be discussed elsewhere in the manuscript.

**Table 1:** Demographic data between groups and in overall sample (ALL‐HD)

	Mean ± SD		
	Pre‐HD (*n* = 12)	Symp‐HD (*n* = 7)	ALL‐HD (*n* = 19)
Demographics			
Gender (M:F)	7:5	5:2	12:7
Age	42.16 ± 12.14 (24, 64)	51.57 ± 8.56 (41, 65)*	46.58 ± 12.25
Education (years)	11.83 ± 2.28	11.57 ± 2.63	11.73 ± 2.35
CAG	42.16 ± 2.72	42.57 ± 1.51	42.31 ± 2.31
ICV	1425.24 ± 170.82	1429.82 ± 120.14	1426 ± 150.50
Time to scan	−40.33 ± 108.73 (−242, 105)	−276.28 ± 70.70 (−364, −174)*	−127.26 ± 150.22 (−364, 105)
Probability of diagnosis	0.21 ± 0.23	–	–
Illness duration	–	1.42 ± 0.78	–
DBS	269.00 ± 104.42	408.92 ± 142.04**	320.55 ± 134.90
UHDRS	0.33 ± 0.65 (0, 2)	17.42 ± 8.32 (11, 33)	6.63 ± 9.75
IQ estimate	112.90 ± 5.48	110.82 ± 7.00	112.13 ± 5.98
BDI‐II	6.75 ± 7.60	3.85 ± 3.57	5.68 ± 6.45
Trails B	73.96 ± 23.63	158.00 ± 94.01**	104.92 ± 70.86
Speeded tapping	215.86 ± 12.65	335.53 ± 100.04**	259.96 ± 83.37
HVLT total recall	24.91 ± 4.29	18.71 ± 9.12	22.63 ± 6.96
HVLT delayed recall	8.75 ± 1.91	7.28 ± 3.63	8.21 ± 23.25
HVLT % retention	87.45 ± 10.46	88.72 ± 37.67	87.92 ± 23.25
HVLT recognition discrimination index	10.25 ± 1.21	9.00 ± 2.70	9.79 ± 1.93
Reaction time (CNV)	820.17 ± 268.94	1272.94 ± 382.69*	971.09 ± 371.63
Volume (mm^3^)			
Right putamen	2692.88 ± 505.79	1673.83 ± 226.60**	2317.44 ± 654.61
Left putamen	2682.02 ± 579.64	1703.18 ± 192.96**	2321.40 ± 673.10
Right caudate	3265.68 ± 662.22	2180.11 ± 199.59**	2865.74 ± 755.45
Left caudate	3208.72 ± 711.01	2115.84 ± 156.22**	2806.08 ± 781.30

CAG, cytosine–adenine–guanine; ICV, intracranial volume. IQ (NART: National Adult Reading Test 2nd Edition). Time to scan computed in days from EEG baseline to MRI. Probability of onset in 5 years calculated from Langbehn et al. (2004). Disease Burden Score (CAG‐35.5) × age; UHDRS, motor subscale score, Unified Huntington's Disease Rating Scale (pre‐HD, UHDRS <5; symp‐HD, UHDRS ≥ 5); predicted Full Scale IQ converted from performance on the National Adult Reading Scale. Nonparametric Whitney–Mann *U* tests for differences between groups; *P <* *0.05; **P <* *0.01.

#### Electrophysiological recording and analysis

Presentation of stimuli and recording of behavioral responses were controlled by Stim2 (Version 4.0; Compumedics, Neuroscan, TX). EEG data were recorded and processed using Scan 4.1 (Compumedics, Neuroscan, Charlotte, NC) software. A 40‐channel Lycra EEG cap with embedded tin surface electrodes was used, with 40 recording electrodes placed according to the international 10/20 system. The EEG was referenced to a point midway between Cz and Pz, with a ground electrode located midway between Fz and Pz. Impedances were below 10 kΩ for all electrodes at the start of the recording. Eye movements (EOG) were measured for subsequent EOG correction, with electrodes placed above and below the left eye, and on the outer canthus of each eye. EEG and EOG signals were amplified using a NuAmps 40‐channel DC amplifier (Compumedics, Neuroscan) with a digital bandpass filter at 0.15–100 Hz, and sampled at 1000 Hz. Data were stored offline for later processing.

#### CNV task

A Go/No‐Go task was employed to elicit electrophysiological components of sensorimotor integration (CNV). All participants were right handed and elected to use their dominant hand during the motor task. A 500‐msec warning stimuli was presented (blue light flash; S1), followed 2.5 sec later by an “X” or “Y” visual cue (S2), appearing randomly on either the right or left side of the screen. Participants were instructed to respond only to the “X” stimulus and to press a button corresponding to its position on the screen. The No‐Go stimuli occurred in 20% of trials, and the intertrial interval was randomly varied between 2500 and 4000 msec across a total of 90 trials.

#### Imaging

Structural MRI images were acquired using a Siemens Magnetom Tim Trio 3 Tesla scanner (Siemens AG, Erlangen, Germany) with a 32‐channel head coil at the Murdoch Children's Research Institute (Royal Children's Hospital, Victoria, Australia). High‐resolution *T*_1_‐weighted images were taken (192 slices, 0.9‐mm slice thickness, 0.8‐mm in‐plane resolution, TE = 2.59 msec, TR = 1900 msec, flip angle = 9°).

### Data analysis

#### Electrophysiological data

The CNV was filtered using a 0.03–35 Hz bandpass zero phase shift filter (24 dB roll‐off) consistent with literature and our previous study (De Tommaso et al. 2007; Turner et al. 2015). The CNV was epoched to the period of −3500 to 1000 msec respective to S2 (Go‐No‐Go) cue presentation. Epochs were baseline corrected (relative to −3500 to −3000 msec) and averaged separately for electrode site. We used automated artifact rejection procedures (±150 μV; excluding EOG channels), accompanied by rejection of contaminated trials by visual inspection. Both Go and No‐Go trials were included in the grand average, and participants with less than 15 epochs were excluded from analysis.

Early and late CNV values were obtained from sites Fz, Cz, and Pz. Selection of electrode sites was made a priori, and aimed to mirror both our previous study (Turner et al. 2015) and prior studies, suggesting possible abnormal activation across these sites (Bartenstein et al. 1997; Ikeda et al. 1997; Weeks et al. 1997; Feigin et al. 2006; Gavazzi et al. 2007; Klöppel et al. 2009). We derived early and late CNVs as peak amplitudes during the epoch −2450 to −2250 msec, and −200 to 0 msec, respectively (De Tommaso et al. 2007). The relative amplitude of the CNV was computed using peak‐to‐peak values which comprised the difference between the peak during the early CNV and that of the late CNV. We used the polyfit function in Matlab™ (MATLAB and Statistics Toolbox, The MathWorks, Inc., Natick, MA) (RRID:SCR_001622) to fit a regression slope for each participant, calculating slope average and goodness of fit. Regression slopes were calculated for main electrode sites Fz, Cz, and Pz.

#### Volume analysis

Neostriatal volumes (caudate and putamen) were obtained by a single trained researcher (F. A. W.) by manual tracing following a validated protocol (intrarater intraclass correlation 0.88–0.98) using ANALYZE 11.0 (Mayo Foundation, Rochester, MI; RRID:SCR_005988) software. Protocol details are available elsewhere (Looi et al. 2008). This manual tracing yields binary shapes for the structures. Intracranial volume (ICV) was calculated using FSL's Brain Extraction Tool (Smith et al. 2002). FSL (RRID:SCR_002823) is freely available for noncommercial use and can be obtained from FMRIB's Software Library (http://fsl.fmrib.ox.ac.uk/fsl/fslwiki/).

#### Shape analysis

Shape analysis procedure replicated a previous study (Macfarlane et al. 2015). Shapes were analyzed in a semiautomated manner using the University of North Carolina shape analysis toolkit (http://www.nitrc.org/projects/spharm-pdm/), full details of which are available elsewhere (Styner et al. 2006; Levitt et al. 2009). Segmented three‐dimensional binaries are initially processed to ensure interior holes are filled, followed by morphologic closing and minimal smoothing. These are then subjected to spherical harmonic shape description, whereby boundary surfaces of each shape are mapped onto the surface of a sphere and the surface coordinates were represented through their spherical harmonic coefficients (Brechbühler et al. 1995). The correspondence between surfaces is established by parameter‐based rotation, based on first‐order expansion of the spherical harmonics. The surfaces are uniformly sampled into a set of 1002 surface points and aligned to a study‐averaged template for each structure (left and right caudate and putamen) using rigid body Procrustes alignment (Bookstein 1996). Scaling normalization was performed to remove the effect of head size/ICV, using a surface scaling factor: *f*_*i*_, where *f*_*i*_ **= (mean [ICV]/ICV *I* ^1/3^) (Styner et al. 2007).

#### Statistical analyses

All statistical analyses were performed using SPSS statistical software version 20.0 (IBM SPSS Statistics, Chicago, IL). The data were initially divided into pre‐HD and symp‐HD samples for the purposes of volumetric comparisons to describe the characteristics of the subgroups. Nonparametric Mann–Whitney *U* tests were used to examine between‐group differences in volume estimates of the left and right caudate and putamen regions. Nonparametric Wilcoxon signed‐rank tests were used to examine within‐group differences in caudate and putamen volumes between hemispheres for each group (pre‐HD, symp‐HD) and the combined group (ALL‐HD).

Correlational analyses for volume and shape (morphology) were conducted on the combined group (ALL‐HD) to increase sample size, statistical power, and account for individual differences in disease progression. All correlational analyses used age, gender, ICV, and time to scan as covariates. For volume, Pearson's partial correlations were used to examine the relationship between volume estimates (left and right caudate and putamen) and electrophysiological, clinical, and behavioral variables. For shape, Pearson's partial correlation was used to determine variable relationships between morphology (left and right caudate and putamen) based on distance of surface points from an average shape (Styner et al. 2006; Levitt et al. 2009). Electrophysiological variables consisted of amplitude, relative amplitude, latency, and slope estimates of the CNV at Fz, Cz, and Pz. Clinical variables included DBS, CAG repeats, and Trails B scores. Behavioral variables included reaction time derived from the CNV task.

## Results

### The CNV

**Figure 2:**
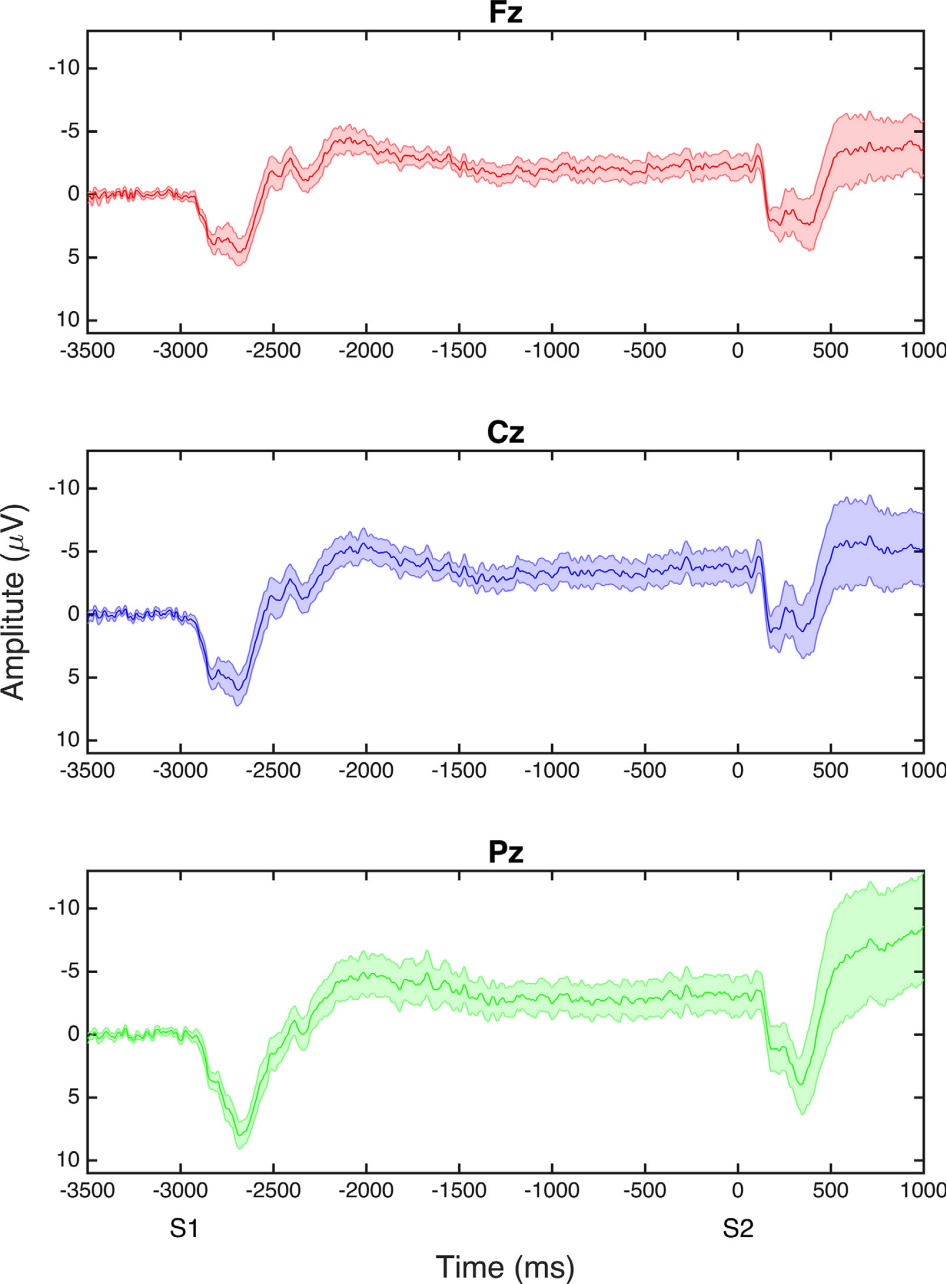
Grand average waveforms of the contingent negative variation (CNV) at Fz, Cz, and Pz; S1 and S2 are the warning and stimulus onset times, respectively. Early CNV refers to the period 550–750 msec following presentation of the warning light (stimulus 1); late CNV refers to the period 200 msec prior to the onset of the Go/No‐Go cue for button press (stimulus 2).

Grand average waveforms of the CNV are shown in Figure 2. Typical grand mean distribution is observed with CNV maximal at central scalp sites (Cz), and beginning approximately 1500 msec prior to presentation of stimulus 2 (S2).

### Volume and shape analyses

Age, gender, and ICV were used as covariates in volume and shape analyses. Symp‐HD demonstrated significantly smaller volumes than pre‐HD of both caudate (right and left *P < *0.001) and putamen (right *P *=* *0.004, left *P *=* *0.010). Nonparametric Wilcoxon signed‐rank tests failed to demonstrate any significant differences in volumes between hemispheres for any group, or the combined group (ALL‐HD). Relationships between neostriatal shape and electrophysiological and behavioral variables were examined; to account for sample size and interindividual variability in disease progression, the pre‐HD and symp‐HD group were combined in analyses to increase statistical power (ALL‐HD).^2^

Volume correlations with CNV latency, amplitude, and relative amplitude are shown in Table 2. For behavioral variables, reaction time was significantly correlated with right putamen volume only (−0.605, *P *=* *0.022). Speeded tapping, a clinical test, was significantly correlated with right caudate volume (−0.540, *P *=* *0.046). Trails B score was significantly correlated with right (−0.593, *P *=* *0.025) and left caudate volumes (−0.555, *P *=* *0.039), as well as the right putamen (−0.540, *P *=* *0.046).

**Table 2:** Partial correlations between caudate and putamen volume and amplitude, relative amplitude (slope), and latency of electrophysiological motor component (CNV)

	Caudate		Putamen	
	Right	Left	Right	Left
Amplitude				
Early				
Fz	−0.424	−0.506	−0.264	−0.269
Cz	−0.340	−0.410	−0.194	−0.151
Pz	0.0709	0.035	−0.088	0.058
Late				
Fz	−0.313	−0.292	−0.387	−0.262
Cz	−0.240	−0.329	−0.015	−0.077
Pz	−0.041	−0.013	−0.344	−0.175
Latency				
Early				
Fz	−0.666**	−0.436	−0.704**	−0.629*
Cz	−0.691**	−0.651*	−0.032	−0.282
Pz	−0.535	−0.614*	−0.275	−0.530
Late				
Fz	−0.685**	−0.747**	−0.314	0.446
Cz	−0.440	−0.547	−0.063	−0.025
Pz	−0.221	−0.405	−0.104	−0.102
Difference/Slope				
Fz	−0.041	−0.039	−0.286	−0.154
Cz	0.049	−0.025	−0.056	−0.169
Pz	−0.286	−0.241	−0.781**	−0.846**

Partial correlations controlled for age, gender, ICV, and time to scan. Time to scan computed in days from EEG baseline to MRI; *N* = 18; df = 12; *P <* *0.05; **P <* *0.01.

**Figure 3:**
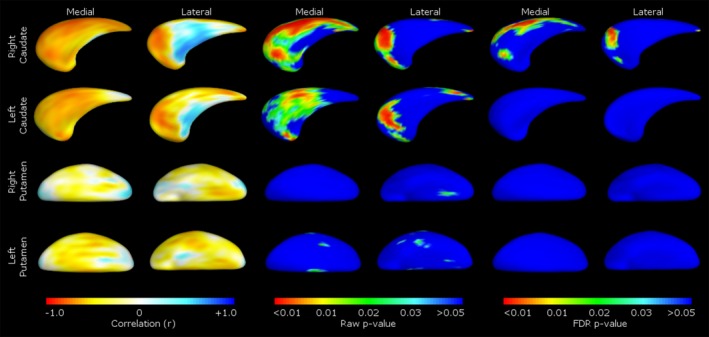
Correlations between caudate and putamen shape and latency of the electrophysiological motor component (early CNV at Cz). Pearson's partial correlations are shown in the left pane, raw *P*‐values in the middle pane, and FDR corrected *P*‐values in the right pane. Medial/lateral denotes viewpoint of the caudate or putamen presented.

**Figure 4:**
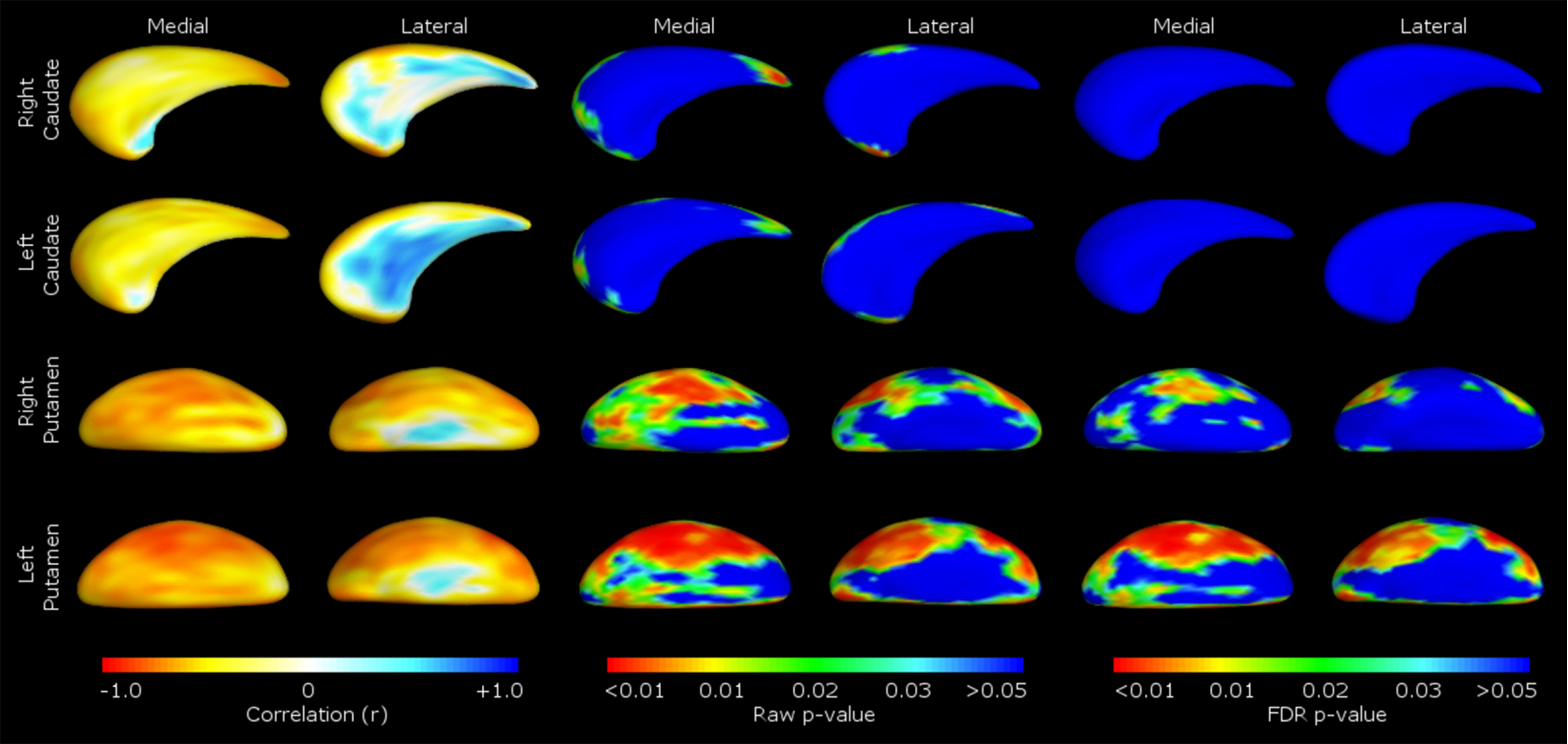
Correlations between caudate and putamen shape and slope of the electrophysiological motor component at Pz. Pearson's partial correlations are shown in the left pane, raw *P*‐values in the middle pane, and FDR corrected *P*‐values in the right pane. Medial/lateral denotes viewpoint of the caudate or putamen presented.

Shape correlations for the early CNV latency at Cz and the CNV slope at Pz are presented in Figures 2 and 3, respectively. Latency of the early CNV component at Cz was negatively correlated with shape in both medial (centralized) and lateral (anterior and posterior; patchy) aspects of the right caudate. Shape deflation was most notable in negative correlations between CNV slope at Pz in the medial and lateral aspects of the right putamen (medial, central, lateral, anterior and posterior), and left putamen (medial and lateral, widespread); most of this shape deflation was seen on the dorsal aspect of the putamen on both medial and lateral sides (Fig. 4).

## Discussion

This study examined the relationship between neural premotor processing during a sensorimotor integration task (CNV) and neostriatal morphology, to better understand the relationship between impaired frontostriatal circuit‐mediated motor function and structural integrity in HD. In these findings, we highlight implications for neural sources of the CNV, and contributions of frontostriatal functional connectivity to premotor performance. All correlational analyses for volume and shape were conducted on the combined group (ALL‐HD) to increase statistical power and account for individual differences in disease progression.

### Volumetric analyses

#### Volume associations with integrity of circuits

Contingent negative variation amplitude across scalp regions and component (early, late) showed no relationship with volume. However, relative activation (slope) of the CNV over parietal regions correlated with right and left putamen volume, with greater slope (indicating appropriate premotor preparation) associated with more volume in these regions. Larger putamen volumes (bilaterally) have been significantly associated with connectivity of M1 with nonmotor regions, including the right precentral gyrus, and the right and left cuneus (Koenig et al. 2014), suggesting that frontostriatal projections to the putamen overlap across multiple, preferential functional network loops. Based on these results, putaminal volume, as an index of structural integrity, seems to be related to the ability to recruit additional compensatory networks. With further decreasing putaminal volumes in symp‐HD, this ability may be lost; larger studies will be needed to confirm this hypothesis.

#### Volumetric associations with timing of premotor activation

Timing of neural premotor activation (latency) was correlated with volume in the right caudate (early Fz, Cz, late Fz), left caudate (early Cz, late Fz) and right and left putamen (early Fz). Additionally, motor execution variables of reaction time and speeded tapping were correlated with volume in the right putamen and caudate, respectively. Lower volumes in these regions, representing decreased structural integrity, were associated with delayed neural premotor activation and execution (slower overall reaction times, fewer taps in speeded trial). Although the groups were combined in morphological analyses, it should be noted that in our previous work, only symp‐HD, but not pre‐HD, demonstrated significantly slower reaction times and fewer taps during the task compared with healthy controls (Turner et al. 2015).

Such volumetric correlations combined with normal task performance (reaction times) may support a threshold of maintained performance prior to significant degeneration, with progressive failure of frontostriatal networks first resulting in heightened and prolonged premotor activation prior to the onset of performance deficits. Indeed, other studies have demonstrated motor performance (tapping variability) correlates with bilateral gray matter atrophy of caudate and putamen, white matter loss, cortical thinning (Bechtel et al. 2010), and approach of symptom onset (Hinton et al. 2007), suggesting degeneration eventually impedes motor precision and possible compensation. In maintaining motor function in the interim, functional specificity of frontostriatal networks and/or compensation is a complex process which likely varies with disease progress. For example, tapping precision in pre‐HD, but not symp‐HD, has been positively correlated with synchrony in the medial primary motor cortex (Poudel et al. 2014), and weakened M1 connectivity with the SMA and dysfunction in the DLPFC using fMRI in pre‐HD, but not symp‐HD (Wolf et al. 2011, 2012). Early structural pre‐HD changes are also suggested to weaken M1 connectivity with premotor regions and the caudate nucleus itself (Unschuld et al. 2012), which may elicit compensation, with decreased caudate and increased SMA and anterior cingulate activation identified in pre‐HD individuals more than 12 years, but not less than 12 years from onset (Paulsen et al. 2004). Within the striatum, motor relay loops connect the M1 and the pallidum (Koenig et al. 2014) as well as premotor regions and the pallidum (Draganski et al. 2008), and thus may circumvent early atrophy. Such temporary recruitment fallible to degeneration is consistent with clinical observation in HD, where by and large pre‐HD individuals do not demonstrate observable motor impairment. This process is likely mediated by individual differences such as cognitive reserve (Papoutsi et al. 2014). These studies highlight the complex nature of motor processing in a degenerative context, with preservation of motor performance dependent upon functional connectivity and available resources.

### Shape analyses

#### Shape associations with integrity of circuits

Shape deflation, representing atrophy of the surface of the striatum, was most notable in negative correlations with CNV slope at Pz (where a flattened slope indicates unregulated premotor activation) corresponding to deflation to the medial and lateral aspects of the right (medial, central, lateral, anterior and posterior) and left putamen (medial and lateral, widespread). This was particularly widespread on the dorsal aspect of the putamen, with greater shape deflation associated with a flatter slope at Pz across the CNV period. Integrity of the putamen is associated with direct motoric functionality (Draganski et al. 2008), with dorsolateral regions responsible for motor and sensorimotor control (Alexander et al. 1986). Successful sensorimotor integration in the CNV requires coordination of motor and nonmotor functionality (planning and execution), with the early component of the CNV theorized to reflect prefrontal and SSMA and the late component prefrontal, M1, S1, temporal, occipital, and SSMA activation (Hamano et al. 1997). Hence, the slope of the CNV may reflect more widespread connectivity of frontostriatal circuits during premotor functions. A flatter Pz slope during CNV may suggest activation is inflexible and does not reflect the demands of the task (early and late), perhaps indicating disruption of premotor circuitry. The link between direct motor functionality and the putaminal integrity (as measured by shape) was supported by negative correlations between reaction time, Pz slope, and volume of the right putamen. Greater atrophy was associated with slower motor execution and flatter slope, respectively. Atrophy to the dorsal putamen may compromise frontal motor projection areas, potentially reflecting a limited ability to recruit dorsolateral circuitry and regulate motoric function.

#### Shape associations with timing of premotor activation

Timing of activation (latency) in the early CNV was related to morphology in both medial (centralized) and lateral (anterior and posterior; patchy) aspects of the right caudate. Greater shape deflation, that is atrophy, in the right caudate was associated with delayed neural premotor activation. Structural integrity of the caudate has been associated with prefrontal motor control; neostriatal motor projections occupy the dorsolateral caudate, connecting premotor and M1 areas and implicating the region in motor control (Haber 2003; Taniwaki et al. 2003). In pre‐HD, structural changes have been associated with weakened connectivity of M1 and premotor regions with the caudate (Unschuld et al. 2012). In older adults, shape deflations in corresponding posterolateral aspects of the left caudate have previously been associated with motor dysfunction in age‐related white matter hyperintensities (Macfarlane et al. 2015). Therefore, based on our results and previous findings, degenerative changes to the (in our case, the right) caudate may impair motor planning and execution, resulting in irregular neural premotor activation and delayed motor execution.

In our previous paper, we highlighted abnormal CNV activation in pre‐HD individuals (Turner et al. 2015). In this follow‐up paper, we aimed to clarify the contribution of the striatum to abnormal premotor activation. Here, we demonstrate that premotor activation is dependent on structural integrity of the striatum (caudate and putamen). We propose that disease‐related atrophy progressively impedes functional connectivity of critical frontostriatal circuits involved in motor control, resulting in subtle premotor changes to the CNV profile. We are not necessarily implying that there is a linear loss of function; however, we have demonstrated there is a correlation of volume loss (atrophy) in the striatum with abnormal CNV at the stages measured. The abnormal CNV consists of delayed and flattened neural premotor activation in the context of normal execution, and from this we draw two conclusions. First, degenerative changes to the right caudate morphology are associated with significantly delayed neural premotor activation and execution, which likely reflects impaired planning and execution of simple movements. Second, atrophy to the dorsolateral putamen is associated with a significantly flattened CNV slope at Pz. Due to circuit organization, progressive putaminal atrophy appears to impede access to crucial dorsolateral circuitry used to regulate motor activation and additional compensatory networks which support motor performance. These results provide strong evidence to support the compromise of various frontostriatal networks through progression of HD, as well as despecialization during early stages of the disease to support motor performance. Taken together, the correlations between the CNV and morphology of the striatum support the utility of concurrent measurement toward localizing neural premotor pathways and developing complimentary biomarkers in HD, which can potentially be implemented in clinical assessment and prognostication.

## Conflict of Interest

None declared.

## Supporting information


**Table S1.** Partial correlations between caudate and putamen volume and amplitude, relative amplitude (slope), and latency of electrophysiological motor component by gene status.

## Notes

1 Supplementary date including electrophysiological data of participants without imaging data (excluded from analysis), along with plots for the combined and paper sample, code for analysis, and raw data for each participant are provided on Figshare (doi: 10.6084/m9.figshare.3168145).

2 A table of partial correlations between groups (pre‐HD and symp‐HD) is included for the reader's interest in Table S1.
