# Supplementary material for: Striatal morphology correlates with frontostriatal electrophysiological motor processing in Huntington's disease: an IMAGE‐HD study
Source: Brain Behav. 2016 Jul 27;6(12):e00511. doi: 10.1002/brb3.511 (PMC5167007; doi:10.1002/brb3.511)
Supplement: Supplementary file 1 — Table S1. Partial correlations between caudate and putamen volume and amplitude, relative amplitude (slope), and latency of electrophysiological motor component by gene status. [file BRB3-6-e00511-s001.docx]

**SUPPLEMENTARY MATERIALS**

**Table 3.** Partial correlations between caudate and putamen volume and amplitude, relative amplitude (slope) and latency of electrophysiological motor component by gene status.

|  | Pre-HD (n = 12) | | | | Symp-HD (n = 6) | | | |
| --- | --- | --- | --- | --- | --- | --- | --- | --- |
|  | Caudate | | Putamen | | Caudate | | Putamen | |
|  | Right | Left | Right | Left | Right | Left | Right | Left |
| Amplitude |  |  |  |  |  |  |  |  |
| *Early* |  |  |  |  |  |  |  |  |
| Fz | -.181 | -.378 | -.115 | -.266 | -.862 | -.980 | -.137 | .932 |
| Cz | -.114 | -.259 | .008 | -.057 | -.769 | -.935 | .026 | .978 |
| Pz | .419 | .345 | .113 | .235 | -.534 | -.779 | .333 | .994 |
| *Late* |  |  |  |  |  |  |  |  |
| Fz | -.084 | .004 | -.424 | -.428 | -.872 | -.984 | -.156 | .925 |
| Cz | -.577 | -.673 | .199 | -.138 | -.439 | -.707 | .433 | .977 |
| Pz | .332 | .376 | -.167 | -.088 | -.319 | -.610 | .545 | .942 |
|  |  |  |  |  |  |  |  |  |
| Latency |  |  |  |  |  |  |  |  |
| *Early* |  |  |  |  |  |  |  |  |
| Fz | -.619 | -.298 | -.637 | -.694 | .726 | .910 | -.090 | -.989 |
| Cz | -.836^**^ | -.728^*^ | .0802 | -.206 | -.960 | -.818 | -.814 | .376 |
| Pz | -.617 | -.701 | -.352 | -.596 | -.874 | -.669 | -.923 | .160 |
| *Late* |  |  |  |  |  |  |  |  |
| Fz | -.458 | -.642 | .0245 | -.310 | -.422 | -.694 | .450 | .973 |
| Cz | .076 | -.260 | .589 | .412 | -.184 | -.493 | .658 | .885 |
| Pz | .236 | -.129 | .327 | .277 | .215 | -.113 | .900 | .632 |
|  |  |  |  |  |  |  |  |  |
| Slope |  |  |  |  |  |  |  |  |
| Fz | -.049 | .0228 | -.375 | -.364 | -.937 | -.772 | -.855 | .306 |
| Cz | -.456 | -.417 | -.614 | -.796^*^ | -.189 | -.497 | .654 | .887 |
| Pz | -.233 | -.058 | -.914^**^ | -.893^**^ | .837 | .615 | .948 | -.0903 |

Partial correlations controlled for age, gender, ICV, and time to scan. Time to scan computed in days from EEG baseline to MRI; N = 18; df = 12; * *p* < .05; ** *p* < .01.
